# Effect of Exposure to Socio-Political Violence and Abuse During Childhood on Executive Planning in Adult Life

**DOI:** 10.3389/fpsyt.2021.693741

**Published:** 2022-02-15

**Authors:** David Andrés Montoya-Arenas, Daniel Londoño-Guzmán, José G. Franco, Ana M. Gaviria

**Affiliations:** ^1^Grupo de Investigación Emoción, Cognición y Conducta, Facultad de Psicología, Universidad Pontificia Bolivariana, Medellín, Colombia; ^2^Grupo de Investigación Psicología y Neurociencias, Facultad de Psicología, Universidad de San Buenaventura, Medellín, Colombia; ^3^Grupo de Investigación en Psiquiatría de Enlace (GIPE), Facultad de Medicina, Universidad Pontificia Bolivariana, Medellín, Colombia; ^4^Grupo de Investigación Psique y Sociedad, Facultad de Ciencias de la Salud, Fundación Universitaria María Cano, Medellín, Colombia

**Keywords:** elderly, child abuse, violence, adverse childhood experiences, executive planning

## Abstract

**Objective:**

The exposure to unfavorable environments during childhood negatively affects the development of the executive planning abilities in adult life. In countries with sociopolitical conflicts, children are exposed to traumatic events as a result of child abuse and sociopolitical violence. The purpose of this study was to analyze the effect of the exposure to both forms of adverse childhood experiences on the executive planning abilities in adults from the general population.

**Method:**

The history of child abuse and sociopolitical violence during childhood was assessed, as well as the executive planning abilities, in 59 adults older than 49 without cognitive impairment or depressive disorder.

**Results:**

Of the sample, 88.1% experienced at least one child abuse event and 47.5% was exposed to sociopolitical violence. Sexual abuse and physical abuse (child abuse) were associated with reduced performance in executive planning. Forced displacement and extortion (sociopolitical violence) had a mixed relationship with planning ability, improving some aspects, and worsening some others. Kidnapping was associated with increased capacity and control of the working memory and executive planning.

**Conclusions:**

The traumatic events during childhood have differential effects on the executive planning skills in the adult life. The exposure to sexual and physical abuse negatively affects executive skills; on the other hand, sociopolitical violence has a mixed or positive impact. Specifically, kidnapping favors the executive planning processes, probably under an evolutionary adaptive mechanism.

## Introduction

Executive planning abilities is important for the autonomy of adults. It is the ability to integrate, sequence, and develop intermediate steps to accomplish goals; it ensures the adaptation to environmental demands, and it favors the organization and direction of behavior, thus determining the functional capacity. Since no behavior is as productive as a planned one, the functionality during adult and old age depends on the integrity of these processes (1).

Executive planning abilities depend on the activation of the prefrontal cortex, responsible for resolution of new and complex situations. The development of these functions follows a gradual pattern that covers critical periods of brain maturation between the ages of 5 and 17 (2). The exposure to abuse or neglect during these ages produces constant modifications in structures and circuits of the nervous central system (CNS), which increases the risk of mental disorders in adults (3). Since the development of these functions follows a slow and progressive course during childhood, the time interval during which they are susceptible to damage due to environmental adversities is wide, with the consequence of compromising the executive planning abilities in subsequent stages (4).

The different forms of child abuse have a differential effect on particular brain regions and circuits. They have been related to, for example, reduced volume in areas of the anterior cingulum, precuneus, parahippocampal gyrus, and hippocampus, regions involved in short- and long-term memory, working memory, planning, sequencing, and monitoring of one's own behavior (5). Specifically, sexual abuse is linked to reduced performance in inhibitory control (6) and verbal memory (7); physical abuse is related to reduced performance in auditory attention and visual-motor integration, problem solving, abstraction, and planning (8); and emotional abuse is connected with perseverative errors (9).

In contrast, some studies have reported a positive effect of adverse early experiences on adult cognition. Ritchie et al. (10) observed that physical and sexual abuse is positively related to verbal fluency and memory. Barnes et al. (11) described that, in the African-American population compared with white population, early adversities, such as food restriction, had a protective effect against cognitive impairment (11). Subsequently Feeney et al. (12) reported similar results, where child sexual abuse history was associated with better overall cognition, memory, executive function, and processing speed. However, most of the research works agree on demonstrating the harmful role of adverse environments during childhood on the cognitive development in adult life (3).

Child abuse has had an exponential growth in the cohorts of children born in the early twentieth century (13). In Latin America, 30% of minors have experienced some form of abuse (14), and in Colombia, its prevalence has been estimated in 36% (15). This abuse involves physical and sexual aggressions of high traumatic intensity, which are recognized as experiences that cause severe stress (10).

Furthermore, in Colombia, there is another source of traumatic experiences during childhood: the extended war situation of varying intensity known as “la violencia” dating back to the 1950s. People who are older than 50 years today were potentially exposed to two forms of trauma (child abuse and extended sociopolitical violence) when they were children (16). A recent study on Colombian adults estimated the prevalence of child abuse above 80%, and the traumatic events associated with sociopolitical violence before the age of 18 reached 13.5% in the case of kidnapping, 17.3% in the cases of forced displacement and extortion, and 20.4% in the case of poverty caused by war (17).

It is not clear yet whether the combination of child abuse and sociopolitical violence during childhood is connected with the decline of cognition during the adult life. It is known that the combination of adverse experiences throughout life with depression or anxiety in adults increases the risk of cognitive impairment and dementia (4); however, it has not been sufficiently studied whether the severity of traumatic childhood events explains a deficient performance in the executive planning of adults.

The purpose of this study was to analyze the effect of the exposure to child abuse and sociopolitical violence during childhood on the executive planning abilities of adults older than 49, controlling for variables such as depressive symptoms, other neuropsychiatric symptoms, and the overall cognitive performance.

The hypothesis is that the severity of the trauma for being exposed to the combination of abuse and sociopolitical violence before the age of 18 causes disturbances in the development of the executive planning ability, associated with poor performance in the adult life.

## Methods

This was a cross-sectional analytical study that analyzes the relationship between the severity of combined exposure to abuse and sociopolitical violence during childhood and executive planning performance, controlling for the effect of neuropsychiatric symptoms, and overall cognitive performance, in a group of adults of the general population from Medellín, Colombia.

### Participants

The sample consisted of 59 men and women older than 49. This age limit was established to ensure that the youngest participant had been born before 1969, time when Colombia was already immersed in the sociopolitical violence dating back to 1948 approximately (16); thus, all the individuals included in the sample grew in Colombia during the period of expansion and consolidation of the violence. In addition, at that age, it is already possible to track changes in the cognitive function due to the effects of aging (18).

According to the curve of prediction for sample size (19), we needed a minimum of 55 participants for the multivariate linear models, with a maximum of 10 predictors within each model, to detect significance for *R*^2^ > 0.250.

Individuals who met the inclusion criteria were identified and, using the snowball technique, they helped locate others with similar characteristics. Most of them were enrolled during 2019 by the members of the Psychology and Neuroscience research group of the Faculty of Psychology of Universidad de San Buenaventura (Medellín), who explained the objectives of the study to potential participants and referred them to the researchers for assessment. This mechanism allowed obtaining a sample that included adults from the general population, older people who attended comprehensive care centers for the elderly and older adults attending nursing homes during the day or who lived there as their regular residence in Medellín (Table 1).

**Table 1:** Demographic, clinical, and cognitive characteristics of 59 older adults from Medellín.

**Sociodemographic characteristics**	
Age in years (M ± SD)	69.08 ± 9.41
Years of formal education (M ± SD)	8.99 ± 5.64
Gender *n* (%)	
Female	25 (42.4)
Male	34 (57.6)
**Clinical characteristics**	
Neuropsychiatric symptoms^a^ *n (%)*	
Delusions	4 (6.8)
Hallucinations	1 (1.7)
Agitation/aggression	5 (8.5)
Dysphoria/depression	11 (18.6)
Anxiety	7 (11.9)
Euphoria/elation	5 (8.5)
Apatdy/indifference	15 (25.4)
Disinhibition	4 (6.8)
Irritability/lability	9 (15.3)
Aberrant motor	–
Nighttime behavior	16 (27.1)
Appetite/eating	7 (11.9)
Depressive symptomatology (GDS)^b^ (M ± SD)	2.44 ± 1.51
Overall cognitive status (MMSE)^c^ (M ± SD)	27.19 ± 1.88
**Outcome measures**	
Executive planning^d^ (M ± SD)	
Total correct moves	10.12 ± 2.56
Total excess moves	10.54 ± 3.74
Latency time	11.36 ± 3.05
Execution time	8.61 ± 3.56
Resolution time	8.69 ± 3.34

a *Cummings Neuropsychiatric Inventory (NPI)*.

b *Total score on tde Yesavage Geriatric Depression Scale (GDS)*.

c *Total score on tde Mini-Mental State Examination (MMSE)*.

d *Values calculated based on tde scaled score of tde Colombian validation of tde Tower of London-Drexel University (TOL-DX)*.

People with cognitive impairment (score < 24 in the Mini-Mental State Examination, MMSE), depressive disorders (score > 5 in the Yesavage Geriatric Depression Scale, GDS), history of alcohol or drug abuse within the 6 months prior to the assessment (clinical interview), and sensory deficits hindering a proper assessment were excluded.

The study was approved by the bioethics committee of Universidad de San Buenaventura, Medellín. All the participants gave their written informed consent after receiving full information.

### Instruments and Measures

#### Sociodemographic and Predictor Variables

We collected sociodemographic data such as gender, age, and years of education in a standard form.

Despite having excluded several psychiatric disorders, the presence of neuropsychiatric symptoms was assessed to control for their interference in the relationship between traumatic childhood experiences and executive planning. To that end, the study used (i) the GDS (20), which, besides depressive disorder, identifies the presence of depressive symptoms in patients without the disorder; according to the exclusion criteria, individuals scoring between 0 and 5 were included in the study; and (ii) the Neuropsychiatric Inventory (NPI) (21), which establishes the presence of 12 symptoms: delusions, hallucinations, agitation/aggression, dysphoria/depression, anxiety, euphoria/elation, apathy/indifference, disinhibition, irritability/lability, aberrant motor, nighttime behavior, and appetite/eating.

We used the total score of the MMSE for assessing the overall cognitive performance (22). It comprises 20 questions related to domains such as spatial and temporal orientation, memory, attention, and language, where higher scores indicate better function, and the maximum score is 30. According to the exclusion criteria, only individuals scoring between ≥24 were included.

The early trauma was assessed using the Colombian version of the Early Trauma Inventory-Self Report (ETI-SRCol) (17). The Inventory is valid and reliable for self-report assessment of traumatic antecedents during childhood (23, 24), and the appropriate culturally adapted versions are amply utilized for measuring early trauma in adult population (25–29). The ETI-SRCol assesses 84 traumatic events (items). It is a self-administered questionnaire that measures, in adults, eight traumatic categories (subscales) before the age of 18. The ETI-SRCol has four subscales for abuse and neglect events: general trauma (31 items), physical abuse (9 items), emotional abuse (7 items), and sexual abuse (15 items); and four subscales for events attributable to sociopolitical violence: displacement (9 items), kidnapping (5 items), extortion (4 items), and poverty (4 items).

Each ETI-SRCol item is dichotomously classified as “existing” or “non-existent,” and the frequency of occurrence of each item is also registered. Therefore, The ETI-SRCol yields to the Childhood Trauma Severity Index (CTSI), a continuous measure that quantifies the total burden of the trauma within each one of the eight subscales of the ETI-SRCol during childhood.

To estimate a CTSI, the frequency of each adverse event is registered on a Likert scale from 1 (once a year) to 6 (more than once a day). The CTSI is calculated by multiplying each positive/existing item by the frequency of the event and then adding these values within each subscale, resulting in a measurement of the trauma severity (24).

In this study, the eight CTSIs represent the predictor variables, as they are a weighted measurement of the severity of the harm caused and are the ones used in multivariate analyses. The sum of all ETI-SRCol “existing” items (ETI-SRCol total score) is reported.

#### Outcome Measures

We used the Tower of London-Drexel University Version (TOL-DX) test to assess the outcome variable, executive planning (30). The TOL-DX examinees should reorganize a series of different colored beads from a start position to a goal position; they must move the beads in three pegs to get the positions indicated by the examiner, following two rules: (1) certain number of beads should remain in each peg and (2) only one bead can be moved at a time. The test consists of 10 items of increasing difficulty with a minimum and maximum acceptable number of moves.

The TOL-DX has five executive planning indicators: (a) Total correct score, which determines the working memory capacity and control; (b) Total moves score, as indicator of the executive planning quality; (c) Total initiation time score, as measurement of the inhibitory control and planning preparation processes; (d) Total execution time score, which indicates the speed or pace at which the plans are put into practice from the start of the execution to its completion or discontinuation; and (e) Total problem-solving time score, which shows the overall speed of executive planning and speed of problem solving.

A scaled score was calculated for each of the five TOL-DX measures with a mean of 10 and a standard deviation of 3, adjusted by age according to the Colombian normative data for the test (31).

### Data Analysis

The demographic, clinical, and neurocognitive characteristics are described through frequencies (percentages) and mean ± standard deviation (SD).

To analyze the relationship of demographic variables (gender, age, and years of education) and clinical variables [neuropsychiatric symptoms (NPI), depressive symptoms (GDS), and overall cognitive performance (MMSE)] with scaled scores in the five TOL-DX executive planning scores, we used the Spearman rho correlation coefficient or the Mann–Whitney *U*-test, as appropriate, reporting, in the latter, the estimated size of the *r* effect.

The Spearman rho coefficient was also used to examine the correlation between the five executive planning scores of the TOL-DX and the CTSI of each subscale of traumatic childhood experiences (ETI-SRCol).

In order to study the relationship between the severity of traumatic childhood experiences and the variation in performance in the five scores of executive planning, controlling for the effect of demographic mediators, clinical mediators, or the overall cognitive performance, five multivariate hierarchical models of robust (bootstrapping) linear regression were adjusted.

Each model included as predictors, in the first step, the demographic, clinical, and/or overall cognitive performance covariates that were significantly associated with the executive planning scores in the bivariate analysis; in the second step, the four CTSIs of the abuse events; and, in the last step, the four CTSIs of the sociopolitical violence events. The eight CTSIs of each subscale were included, because it is feasible for an individual to be simultaneously exposed to different forms of abuse and violence. The scaled score of each executive planning score of the TOL-DX was the outcome or dependent variable in each model.

This hierarchical strategy was chosen to observe the effect on the variability of executive planning, by introducing first those variables that have been tested in other studies, then the abuse variables already studied, and lastly the traumatic events caused by the violence; thus, it is possible to control the additive effect of covariables and both forms of childhood trauma.

The simultaneous entry method was used for all the predictor variables in the models. The continuous variables were kept in their original form, and if any categorical variables were included, these were always dichotomous. Since the values of the continuous variables violated the assumptions of linear regression, wild bootstrap was applied with 2,000 samples, and 95% confidence intervals with bias correction (BCa) were derived for each beta coefficient in the final model.

The significance level of *p* < 0.05 was assumed, and statistical tests were two-tailed. Data were analyzed on SPSS-22.0.

## Results

**Figure 1:**
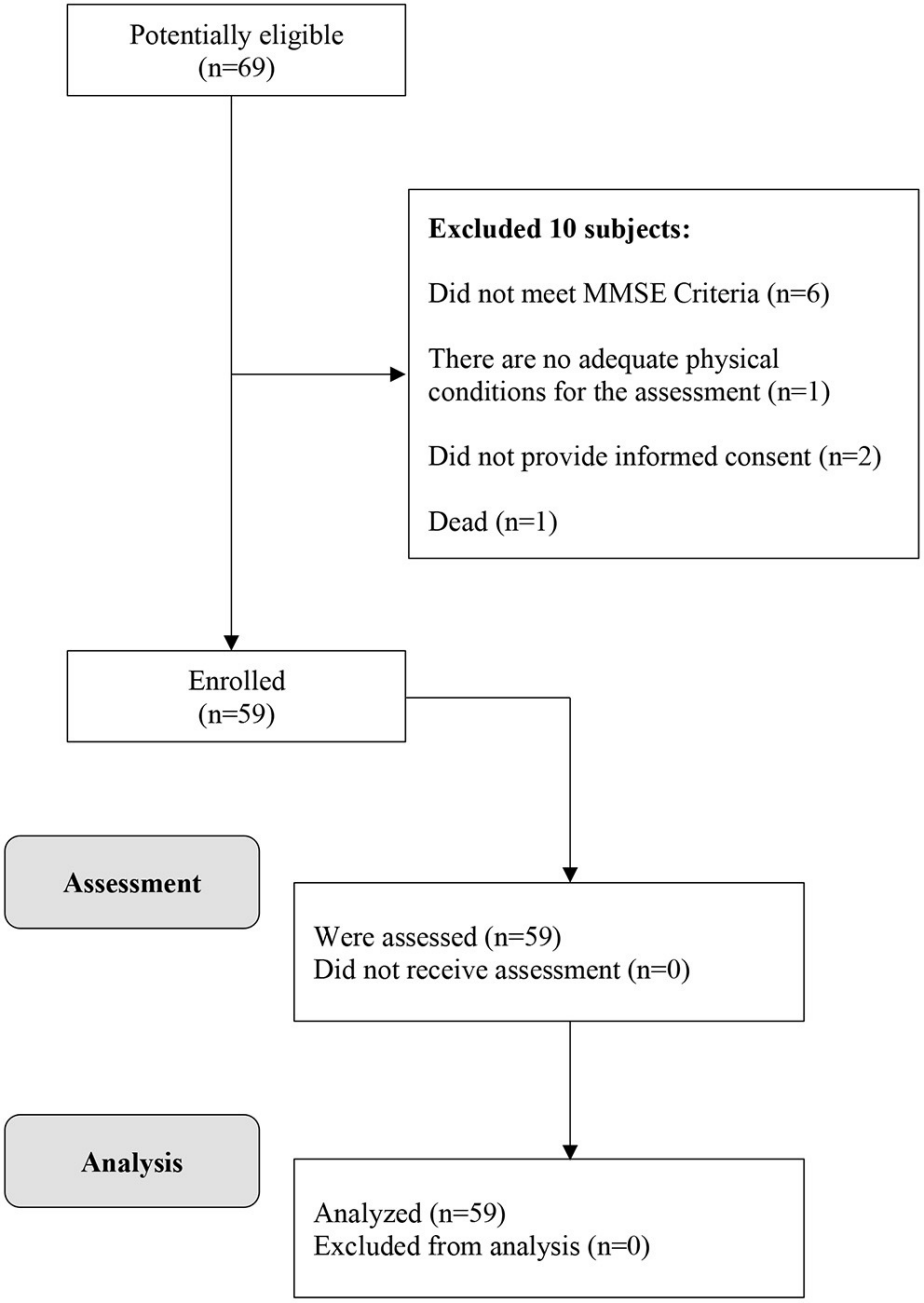
Flow diagram of the study participants.

The diagram of the participants is shown in Figure 1.

### Characteristics of the Sample and Frequency of Traumatic Childhood Experiences

The sample of 59 participants was composed mostly of men (57.6%), with average age of 69.08 ± 9.41 and a mean of 9 ± 5.6 years of education. Despite having excluded individuals with depressive disorder, mood symptoms such as sleep disturbances (27.1%), apathy/indifference (25.4%), dysphoria/depression (18.5%), and irritability/lability (15.3%) were found. Regarding performance in the five executive planning scores, the means of the scaled scores are within the Colombian normative range (M = 10 ± 3) for age, with the total execution time and total problem-solving time scoring the lowest (Table 1).

Regarding traumatic childhood experiences, the total score of the ETI-SRCol was 10.05 ± 6.76. The 94.4% of participants experienced at least one adverse childhood event and, of these, 88.1% were related to experiences of abuse and/or neglect. The 37.3% mentioned having been exposed to three or more experiences of physical abuse, while 44% were victims of any form of sexual abuse (Table 2).

**Table 2:** Frequency of traumatic events during childhood (ETI-SRCol items) grouped in eight categories (subscales) and severity of each subscale (Childhood Trauma Severity Index) in 59 adults from Medellín, Colombia.

**Early traumatic**	**Frequency**	**Childhood trauma severity**	
**experiences**		**index (CTSI)**	
	***n* (%)**	**M ±SD**	**Range**
General trauma			
1	9 (15.3)	7.37 ± 7.35	0–38
2	7 (11.9)		
≥3	36 (61)		
Physical abuse			
1	14 (23.7)	6.13 ± 7.10	0–27
2	9 (15.3)		
≥3	22 (37.3)		
Emotional abuse			
1	9 (15.3)	6.05 ± 5.80	0–22
2	11 (18.6)		
≥3	20 (33.9)		
Sexual abuse			
1	13 (22)	1.81 ± 3.42	0–18
2	8 (13.6)		
≥3	5 (8.5)		
Displacement			
1	4 (6.8)	0.44 ± 1.64	0–10
2	1 (1.7)		
≥3	1 (1.7)		
Kidnapping			
1	2 (3.4)	0.10 ± 0.54	0–4
2	–		
≥3	2 (3.4)		
Extortion			
1	2 (3.4)	0.06 ± 0.31	0–2
2	1 (1.7)		
≥3	–		
Extreme poverty			
1	8 (13.6)	2.23 ± 4.09	0–15
2	6 (10.2)		
≥3	9 (15.3)		
Total ETI-SRCol			
1	2 (3.4)	24.22 ± 19.78	0–89
2	2 (8.5)		
≥3	49 (83.1)		

As for the types of sociopolitical violence, 47.5% (*n* = 28) reported having been exposed at least once before the age of 18, the most frequent being in poverty as a consequence of violence (39.1%) (Table 2).

### Bivariate Association of Demographic and Clinical Characteristics With Executive Planning Ability Scores on the TOL-DX

Women performed significantly better on TOL-DX total initiation time score (*Mdn* = 13; *RI* = 3) compared to men (*Mdn* = 10 *RI* = 5), *U* = 251, *z* = −2.68, p = 0.007, *r* = −0.34. Regarding overall cognitive performance, a direct and significant relationship was found between the MMSE score and the scores in total correct (*r*_*s*_ = 0.317 *p* = 0.014) and total problem-solving time scores (*r*_*s*_ = 0.279 *p* = 0.032) (Table 3).

**Table 3:** Spearman correlation coefficient (and significance levels) between potential mediators, traumatic childhood experiences, and TOL-DX outcome measures in 59 adults from Medellín, Colombia.

	**Total correct moves^a^**	**Total excess moves^a^**	**Latency time^a^**	**Execution time^a^**	**Resolution time^a^**
Age (years)	−0.086	0.109	−0.160	0.091	0.008
Education (years)	−0.052	−0.230	0.243	−0.011	0.026
GDS^b^	−0.036	0.002	−0.214	−0.066	−0.129
MMSE^c^	0.317^*^	0.117	0.168	0.221	0.279^*^
**Childhood trauma severity index (CTSI)** ^d^						
General trauma CTSI	0.041	0.023	−0.202	−0.198	−0.151
Physical abuse CTSI	−0.106	−0.150	−0.236	−0.174	−0.148
Emotional abuse CTSI	−0.207	−0.148	–0.323^*^	−0.131	−0.135
Sexual abuse CTSI	−0.030	–0.312^*^	−0.080	−0.158	−0.115
Displacement CTSI	0.008	−0.155	−0.027	−0.094	−0.082
Kidnapping CTSI	0.289^*^	0.197	−0.165	0.193	0.181
Extortion CTSI	0.111	0.135	−0.190	0.088	0.092
Poverty CTSI	−0.040	−0.032	−0.126	0.168	0.177
Total CTSI	−0.132	−0.141	–0.347^**^	−0.226	−0.199

a *Tower of London-Drexel University (TOL-DX)*.

b *Yesavage geriatric depression scale*.

c *Mini-mental state examination*.

d *Early trauma inventory (ETI-SRCol)*.

* *p < 0.05*;

** *p < 0.01*.

When assessing the influence of neuropsychiatric symptoms on the executive planning abilities, we found that people with agitation/aggression symptoms obtained lower total execution time score (*Mdn* = 6 *RI* = 5) compared to those who did not present these symptoms (*Mdn* = 10 *RI* = 4), *U* = 45, *z* = −2.47, *p* = 0.013, *r* = −0.32. The same symptomatic group also scored significantly lower on total problem-solving time score (*Mdn* = 6 *RI* = 5) compared to the group without agitation/aggression symptoms (*Mdn* = 10 *RI* = 4), *U* = 53, *z* = −2.25, *p* = 0.024, *r* = −0.33. Lastly, the presence of exaltation symptoms, i.e., euphoria/elation, was associated with lower performance in the total initiation time score (*Mdn* = 9 *RI* = 7) compared to the absence of these symptoms (*Mdn* = 12 *RI* = 4), *U* = 62.5, *z* = −1.98, *p* = 0.047, *r* = −0.24. No other significant association was found for neuropsychiatric symptoms and executive planning (non-significant comparisons not shown).

### Multivariate Models of the Relationship of Childhood Trauma With the Executive Planning Scores

Table 4 shows the last steps of the five models of the relationship between the severity of childhood traumas and TOL-DX executive planning scores as dependent variables.

**Table 4:** Variables in the final multiple linear regression model for executive planning indices, with bias corrected and accelerated 95% confidence intervals (95% CI).

	***b* (95% CI)**	**SE B**	**Beta**	***p*-value**
**Total correct moves**				
(Constant)	0.48 (−6.96, 8.35)	3.59		0.917
MMSE	0.37 (0.13, 0.59)	0.13	0.27	0.017
General trauma CTSI	0.07 (−0.01, 0.15)	0.04	0.20	0.172
Physical abuse CTSI	−0.06 (−0.15, 0.02)	0.04	−0.17	0.194
Emotional abuse CTSI	−0.09 (−0.22, 0.02)	0.06	−0.21	0.188
Sexual abuse CTSI	−0.02 (−0.17, 0.14)	0.08	−0.02	0.855
Displacement CTSI	−0.30 (−0.73, 0.13)	0.25	−0.19	0.295
Kidnapping CTSI	1.49 (0.66, 2.38)	0.43	0.32	**0.001**
Extortion CTSI	0.51 (−1.14, 2.11)	0.90	0.06	0.596
Poverty CTSI	0.05 (−0.10, 0.19)	0.07	0.08	0.548
*R*^2^ = 0.092 for step 1; Δ*R*^2^ = 0.056 for step 2 (*p* = 0.49), Δ*R*^2^ = 0.121 for step 3 (*p* = 0.10); *F* = 2.00 *p* = 0.059; Durbin-Watson = 2.33				
**Total excess moves**				
(Constant)	11.11 (9.19, 13.52)	0.88		0.000
General trauma CTSI	0.05 (−0.06, 0.15)	0.06	0.10	0.395
Physical abuse CTSI	0.01 (−0.12, 0.13)	0.07	0.03	0.827
Emotional abuse CTSI	−0.01 (−0.15, 0.10)	0.08	−0.02	0.901
Sexual abuse CTSI	–0.37 (–0.72, –0.04)	0.18	–0.34	0.034
Displacement CTSI	–0.69 (–1.29, –0.13)	0.34	–0.30	0.017
Kidnapping CTSI	2.29 (1.01, 3.71)	0.64	0.33	0.003
Extortion CTSI	3.27 (0.79, 5.46)	1.30	0.27	0.007
Poverty CTSI	−0.21 (−0.46, 0.03)	0.13	−0.22	0.125
*R*^2^ = 0.08 for step 1; Δ*R*^2^ = 0.19 for step 2 (*p* < 0.05); *F* = 2.35 *p* < 0.05; Durbin-Watson = 1.87				
**Latency time**				
(Constant)	11.91 (10.11, 14.45)	0.92		0.000
Sex (0 = Man; 1 = Woman)	1.83 (0.65, 2.68)	0.71	0.30	0.012
Euphoria (0 = No; 1 = Yes)	–2.64 (–4.18, –1.34)	0.83	–0.24	0.001
General trauma CTSI	0.08 (−0.01, 0.16)	0.05	0.19	0.105
Physical abuse CTSI	–0.16 (–0.26, –0.07)	0.05	–0.37	0.004
Emotional abuse CTSI	−0.07 (−0.18, 0.02)	0.06	−0.14	0.224
Sexual abuse CTSI	−0.10 (−0.23, 0.03)	0.07	−0.11	0.126
Displacement CTSI	0.30 (0.06, 0.54)	0.14	0.16	0.007
Kidnapping CTSI	0.41 (−0.28, 1.10)	0.33	0.07	0.204
Extortion CTSI	–3.70 (–5.11, –2.34)	0.76	–0.38	0.000
Poverty CTSI	−0.02 (−0.18, 0.13)	0.08	−0.02	0.855
*R*^2^ = 0.21 for step 1; Δ*R*^2^ = 0.11 for step 2 (*p* = 0.10), Δ*R*^2^ = 0.14 for step 3 (*p* < 0.05); *F* = 4.18 *p* < 0.001; Durbin-Watson = 1.91				
**Execution time**				
(Constant)	9.86 (8.17, 12.41)	0.82		0.000
Agitation/aggression (0 = No; 1 = Yes)	–4.39 (–6.25, –2.73)	1.05	–0.34	0.000
General trauma CTSI	−0.03 (−0.14, 0.07)	0.06	−0.06	0.630
Physical abuse CTSI	−0.09 (−0.21, 0.02)	0.07	−0.17	0.225
Emotional abuse CTSI	−0.00 (−0.12, 0.08)	0.06	−0.01	0.939
Sexual abuse CTSI	–0.31 (–0.57, –0.08)	0.14	–0.30	0.028
Displacement CTSI	−0.31 (−0.90, 0.28)	0.35	−0.14	0.467
Kidnapping CTSI	1.47 (0.28, 2.74)	0.62	0.23	0.018
Extortion CTSI	2.59 (0.06, 4.97)	1.34	0.23	0.056
Poverty CTSI	0.08 (−0.18, 0.34)	0.15	0.10	0.679
*R*^2^ = 0.10 for step 1; Δ*R*^2^ = 0.11 for step 2 (*p* = 0.12), Δ*R*^2^ = 0.13 for step 3 (*p* = 0.06); *F* = 2.80 *p* < 0.05; Durbin-Watson = 1.79				
**Resolution time**				
(Constant)	−2.61 (−18.52, 18.95)	7.47		0.782
MMSE	0.45 (0.05, 0.68)	0.25	0.25	0.134
Agitation/aggression (0 = No; 1 = Yes)	–3.49 (–5.98, –1.39)	1.33	–0.29	0.005
General trauma CTSI	−0.01 (−0.12, 0.09)	0.06	−0.02	0.864
Physical abuse CTSI	−0.07 (−0.20, 0.05)	0.07	−0.16	0.324
Emotional abuse CTSI	−0.01 (−0.13, 0.09)	0.07	−0.01	0.920
Sexual abuse CTSI	−0.22 (−0.47, 0.00)	0.12	−0.23	0.105
Displacement CTSI	−0.21 (−0.74, 0.29)	0.31	−0.10	0.644
Kidnapping CTSI	0.77 (−0.54, 2.24)	0.63	0.13	0.267
Extortion CTSI	1.40 (−0.80, 3.53)	1.13	0.13	0.269
Poverty CTSI	0.16 (−0.12, 0.40)	0.15	0.19	0.354
*R*^2^ = 0.16 for step 1; Δ*R*^2^ = 0.07 for step 2 (*p* = 0.35), Δ*R*^2^ = 0.09 for step 3 (*p* = 0.21); *F* = 2.16 *p* < 0.05; Durbin-Watson = 1.99				

*95% CIs and standard errors (SE) based on 2000 wild bootstrap samples*.

After controlling for significant covariates in the bivariate analysis, the CTSI indexes of the ETI-SRCol related to traumatic abuse events, i.e., sexual and physical abuse, were associated with lower executive planning performance. Specifically, sexual abuse was associated with poorer performance in total moves score and total execution time score, while physical abuse was linked, also inversely, to the total initiation time score.

Sociopolitical violence indexes showed a complex association pattern with executive planning. Displacement and extortion CTSIs were associated with lower scores in total moves and total initiation time, respectively. Conversely, these two same CTSIs, displacement and extortion, were associated with better performance in total initiation time score and total moves score, respectively. Kidnapping was linked to better performance in total correct score, total moves score, and total execution time score (Table 4).

## Discussion

This study investigated the effects of traumatic childhood experiences on the executive planning abilities in adults without cognitive impairment or depression. Besides analyzing exposure to traumatic experiences related to child abuse, we also addressed the effect of traumatic events caused by sociopolitical violence in early neurodevelopmental stages. The results of this research are innovative because, to our knowledge, this is the first study to explore the severity, which considers perpetuation in time, of traumatic events related to prolonged sociopolitical violence of varying intensity in childhood, combined with the typical forms of child abuse and their effect on cognition in adulthood.

A significant part of the sample (88.1%) was exposed to severe abuse/neglect by people who had a role of responsibility, trust, or power over them. These results are consistent with data on the prevalence of traumatic childhood experiences in adults (10). Likewise, almost half of the participants (47.5%) expressed having been exposed, before the age of 18, to some kind of violence perpetrated by armed groups whose acts were justified by political, economic, or social reasons. These findings coincide with those of a study with a sample of Colombian adults (17). Moreover, the score mean of 10 in the ETI-SRCol also reflects the high exposure to abuse and violence during childhood.

In the multivariate models, as expected, symptoms of euphoria/elation and agitation/aggression affected the inhibitory control capacity, the executive planning speed, and the pace for problem-solving plan implementation (Table 4). To understand the above, it is necessary to consider that increased motor activity, aggression, and euphoria are part of a global hyperactivity manifestation typical of dysfunction of the orbitofrontal cortex, the anterior cingulate region, the insula, and the frontotemporal junction (32). We also found that the women in this study performed better than men in inhibitory control and planning preparation, something that has already been discussed in the literature (33). Finally, a positive relationship was found between overall cognitive performance and working memory capacity and control.

According to the multivariate models, traumatic childhood experiences affected the executive planning abilities. Severe abuse events, especially those related to neglect and physical and sexual abuse, had negative impact on the quality of executive processes. In addition, experiences related to violence had a complex pattern, since some types of trauma had a mixed (positive and negative) effect on various aspects of planning, and others had a beneficial impact on several processes.

### Negative Effects of Child Abuse on the Planning Ability

Sexual abuse was associated with lower executive planning quality, thus affecting the speed or pace at which executive plans are implemented. Similarly, physical abuse negatively impacted the inhibitory response processes and the planning preparation.

Similar cognitive patterns have been observed in previous studies on severe forms of this type of abuse, where exposure to these stressors was linked to deficient cognitive performance (4, 34). Specifically, a history of repeated sexual abuse is associated with decreased working memory performance (35).

Analyzing the impact of the severity of traumatic events on cognition allows a better estimate of the repercussions of early adversity during the brain development stages and the long-term impact on cognitive function. Severe stress due to adverse childhood events is known to cause changes in the hypothalamic-pituitary-adrenal (HPA) axis, resulting in high glucocorticoid levels that affect the hippocampal development, which in the long term result, for example, in verbal declarative memory deficits (36).

In this study, the perpetuation of early abuse negatively impacted the executive planning abilities. This association may also be explained by the allostatic load hypothesis (i.e., the adaptive capacity to maintain homeostasis in response to stressors), which reveals how stress responses promote abnormal neurodevelopment (37), generating a pattern of increased stress and adversity throughout life, the cumulative effects of which may contribute to poor executive performance.

Another possible explanation is that adverse events lead to lower cognitive performance throughout life, given that—as it can be observed in the sample—these children often have fewer years of education and socioeconomic opportunities (38), resulting in lower cognitive reserve. However, in this study, the years of education or poverty conditions in childhood were not directly related to executive planning abilities, coinciding with other studies that have found a slight effect of this variable, in contrast to the direct relationship of early traumatic experiences (38, 39).

It is worth noting that there is evidence contrary to the relationship established in this study between child sexual abuse and cognition in adults. The work by Ritchie et al. (10) and Feeney et al. (12) describe a positive effect on some aspects of the information processing, which suggests the need to continue delving into more ample explanatory models on the role of distal neurodevelopmental events on adult cognition.

### Mixed Effects of Sociopolitical Violence Events on the Planning Ability

Two types of sociopolitical violence showed a mixed pattern of relationship with the planning ability in the multivariate models, forced displacement, and extortion.

Displacement was associated with lower executive planning quality and, at the same time, was related to better inhibitory control and planning preparation. Conversely, extortion implied better executive planning quality, but it was associated with poorer inhibitory response and planning preparation.

Studies with children displaced by violence in Colombia have established that forced migration at early ages affects cognitive and emotional development, triggering a feeling of insecurity (40). It is possible that the mechanisms of neurodevelopmental damage due to physical and sexual abuse are similar to those of displacement.

Extortion implies that children are directly or indirectly threatened and coerced over a prolonged period of time. Being exposed during childhood to permanent threat of harm in contexts with a high probability of abuse reduces the overall cognitive performance during adulthood (41).

Regarding the beneficial effects of extortion and displacement on cognition, two explanatory hypotheses, not mutually exclusive, are to be considered. In the first place, the implementation of adaptive mechanisms weakens the effect of the trauma over time (12). These mechanisms would increase neuronal plasticity in structures of the prefrontal cortex, also promoting the cognitive reserve. In the second place, as part of the emotional response, traumas activate the amygdala, which favors the consolidation of memory and the processing of threatening stimuli, with consequent cognitive training to better respond to environmental demands (42).

### Positive Effects of Sociopolitical Violence Events on the Planning Ability

An interesting finding was the positive effect of kidnapping on the planning ability. This event, characterized by being considerably prolonged in the Colombian case, was associated with better capacity and control of the working memory, quality of the executive planning, and speed at which executive plans are implemented.

These findings suggest that this situation, known for causing prolonged stress and the need to constantly search for solutions, activates resilience mechanisms that influence individuals' ability to adapt and thrive after exposure to trauma, leading to reduced vulnerability to cognitive impairment, probably thanks to neuronal compensatory processes (10, 12).

A study with African Americans reported comparable findings. Adverse factors involving long-term stress and solution seeking, such as food restrictions in childhood, were associated with a slower rate of cognitive decline (11).

The findings of this study could also be explained from an evolutionary perspective, since surviving to advanced age despite early adversity could be an indicator of evolutionary advantages that make individuals more resistant to adversity itself (43). Such advantages would also be demonstrated by better executive skills, which would have favored individuals' recovery from the effects of an adverse childhood.

### Limitations, Strengths, and Conclusions

This study has several limitations. The first one is that the retrospective assessment of traumas may lead to underestimating their prevalence; however, the use of an inventory adapted to and validated with a standardized list of traumatic experiences improves the measurement reliability to determine the exposure to and severity of traumas (44). Additionally, some studies have suggested that trauma memories are reliable, especially in people without depressive disorder (such as in this sample) because possible retrospective falsifications are minimized (45).

Another limitation is that posttraumatic stress symptoms were not measured after the traumatic experience or in adult life; therefore, it is not possible to know clearly whether the lower executive planning performance is associated with the early trauma or is a result of posttraumatic stress disorder. However, given that the CTSI was analyzed for each trauma subscale, this limitation may have been overcome. The severity index measures the total trauma burden, better reflecting the long-term effect of exposure to the traumatic events analyzed.

Since we used a non-probability sample, caution should be exercised when generalizing data. On the other hand, this is the first study assessing, besides the classic child abuse, the severity of situations of conflict and sociopolitical violence in a sample from the general population, with clear inclusion and exclusion criteria.

The study has some strengths. As far as we know, this is the first report on the long-term impact of early exposure to traumatic events related to sociopolitical violence (in the context of a prolonged conflict of varying intensity) on cognitive performance, specifically on executive planning. The simultaneous analysis of traumatic experiences associated with abuse and sociopolitical violence offers a broader spectrum of events that (negatively and positively) affect the neurodevelopment and the adaptive capacity, the effects of which can be traced in adult life.

In short, traumatic childhood events exert differential effects on the executive planning abilities. Early exposure to physical and sexual abuse has a negative impact on the executive skills in adulthood, while some traumatic events related to a long-lasting environment of sociopolitical violence, particularly kidnapping, favor the refinement of executive planning processes, presumably as a mechanism of evolutionary adaptation.

## Data Availability Statement

The datasets presented in this article are not readily available because the participants have not given consent to have their data publicly stored, however, de-identified data are available upon request. Requests to access the datasets should be directed to Ana M. Gaviria, amigago@gmail.com.

## Ethics Statement

The studies involving human participants were reviewed and approved by the Bioethics Committee of Universidad de San Buenaventura. The patients/participants provided their written informed consent to participate in this study.

## Author Contributions

DM-A oversaw study design and completion of project, with assistance of DL-G. DL-G conducted neuropsychological evaluations. JF oversaw data analysis and manuscript completion. AG oversaw study design, completed all data analyses, and writing of manuscript. All authors contributed to the article and approved the submitted version.

## Funding

This work was internally financed by the Centro de Investigación para el Desarrollo y la Innovación (CIDI) from the Universidad Pontificia Bolivariana, the Dirección de Investigaciones from the Universidad de San Buenaventura, and the Centro de Investigaciones y Desarrollo Empresarial (CIDE) from the Fundación Universitaria María Cano. The funders had no role in study design, data collection and analysis, decision to publish, or preparation of the manuscript.

## Conflict of Interest

The authors declare that the research was conducted in the absence of any commercial or financial relationships that could be construed as a potential conflict of interest.

## Publisher's Note

All claims expressed in this article are solely those of the authors and do not necessarily represent those of their affiliated organizations, or those of the publisher, the editors and the reviewers. Any product that may be evaluated in this article, or claim that may be made by its manufacturer, is not guaranteed or endorsed by the publisher.
